# Evaluation of the general health of the infertile couples 

**Published:** 2011

**Authors:** Mohammad Hossein Baghiani Moghadam, Amir Hosein Aminian, Ali Mohammad Abdoli, Najmeh Seighal, Hosein Falahzadeh, Nasrin Ghasemi

**Affiliations:** 1 Department of Health Education, Faculty of Health, Shahid Sadoughi University of Medical Sciences, Yazd, Iran.; 2 Department of Psychology, Consultation Center, Shahid Sadoughi University of Medical Sciences, Yazd, Iran.; 3 Research and Clinical Centre for Infertility, Shahid Sadoughi University of Medical Sciences, Yazd, Iran.; 4 Department of General health, Faculty of Health, Shahid Sadoughi University of Medical Sciences, Yazd, Iran.; 5 Department of Medical Genetics, Research and Clinical Centre for Infertility, Shahid Sadoughi University of Medical Sciences, Yazd, Iran.

**Keywords:** *GHQ*-*28*, *Infertile couples*, *Sub*- *scales of GHQ*

## Abstract

**Background:**

The prevalence of infertility is variable between 5-30% around the world. In Iran, more than 2 million couples suffer from infertility. Infertility causes depression, anxiety, social isolation and sexual dysfunction.

**Objective:**

This descriptive study was undertaken to determine general health in infertile couples.

**Materials and Methods:**

One hundred and fifty infertile couples attending Yazd Research and Clinical Center for Infertility were randomly selected during March till September 2009. The GHQ-28 questionnaires were completed by researchers, based on face to face interview. It contains 28 items, which have been divided to four sub- items. The results come out by scoring the patients answer from 0 to 84. All data were analyzed by Chi-square and t-test in SPSS software.

**Results:**

GHQ scores of all sub-scales and total in women were more than men, which shows general health condition in women is worse than men. There was no relation between the duration of infertility and general health scores.

**Conclusion:**

This study suggests that the infertility has significant effect (p=0.001) on health situation of infertile couples, especially infertile women. They are at risk of somatic symptoms (p=0.0001), social dysfunction (p=0.0001) and severe depression (p=0.0001). GHQ could provided help and support in order to improve the health situation of infertile couples.

## Introduction

Infertility causes a big social distress and it is accompanied by numerous psychological and social problems. Sub fertility affects 10-15% of individual in western world (1). The prevalence of infertility in the different countries is variable between 5-30% and in Iran more than 2 million infertile couples were found (2). Infertility is one of the major crises of life (3), which could cause depression, anxiety, social isolation and sexual dysfunction (4, 5). Infertile couples are more anxious and emotionally distressed than other fertile people (6), which stated by WHO (7). 

This psychiatric problem might either be the cause of infertility or the consequence of it (6). For many couples, infertility is undeniably a major life crisis and psychologically stressful (9, 10), so infertility is known as an agonizing and disappointing stressor for couples. Infertility could affect personal, social and marital relationships, and might cause mental instability and lead to divorce. Previous study suggests that infertility is more stressful for women than men (11-15). 

Women with idiopathic infertility are more anxious and have lower self esteem and life-style than others, but also reported greater marital unsatisfaction and greater unsatisfaction in other areas of life than controls (16-18). Even women in assisted reproductive technique (ART) treatment, experienced more emotional and social problems than same age fertile women (19). Couples seek ART treatment, might have numerous psychological disorders. According to Iranian culture and the high birth rate in Iran, psycho-social aspects of infertility are very important especially for women. It effects their quality of life, which could refers to the patients' physical condition as a consequence of disease or the treatment or psychological aspect reflects the patient's satisfaction with social roles and activities. The psychological aspect refers mostly to anxiety and depression (20-22). 

In order to assess the psychological aspect of quality of life, the 28-item version of the General Health Questionnaire (GHQ-28) could be used (23, 24). This self-report questionnaire was designed for detection of current psychiatric disorder. The GHQ was developed in a few countries, and it had been translated into about 38 languages with over 50 validity studies (23-28).

This descriptive study was undertaken to determine general health in infertile couples using GHQ-28.

## Materials and methods

This descriptive study evaluated general health of 150 infertile couples attending to the Yazd Research and Clinical Centre for Infertility, which were randomly selected during March to September 2009. The data were gathered by the researchers, based on face to face interview. 

The questionnaires contain two sections; first is a demographic information and second is GHQ-28. GHQ-28 is used to detect psychiatric disorder in the general population and within community or non-psychiatric clinical settings such as primary care or general medical outpatients. This self-report questionnaire was designed for detection of current psychiatric disorder. 

The GHQ was developed in a few countries during 1960s and 1970s. Goldberg and Williams, in 1988, reported that GHQ had been translated into about 38 languages, and over 50 validity studies have been published. However, these validity studies were conducted mainly in Western European countries and the USA (23-28). They compare their recent psychological state with usual state and it is sensitive for short-term psychiatric disorders. All items have a 4 point scoring system using Likert scoring (0-1-2-3). GHQ-28 divided into four scales, which have been contains 28 queries. The four scales are as follow: somatic symptoms (item 1-7), anxiety/insomnia (8-14), social dysfunction (items 15-21) and depression (items 22-28).

There is no threshold for individual sub- scales. Individual sub-scales are used for providing individual diagnostic or profile information. For identifying general health condition with GHQ-28, the total of the sub- scales was used. In the GHQ-28 the respondent is asked to compare his recent psychological state with his usual state. For each item four possible answer are available [1) not at all, 2) no more than usual, 3) rather more than usual, and 4) much more than usual]. Scoring from 0 to 3 is applied and the total scale score is from 0 to 84. Every sub-scales are composed of 4 points Likert scales.

The minimum and maximum scales of every sub-scales are 0 to 21. In total the participants with scores of lower than 23 have normal general health and participants with scores more than 23 are not healthy. Participants with sub-scale scores lower than 7, are healthy, scores between 7 and 14 are moderately healthy and with score more than 14 not healthy. Previous researches in Iran reported that the validity of GHQ questionnaire are very high. Yaghoby reported the sensitivity and specificity of it %89.5 and %82 respectively (29) and Palahang reported the reliability of it 91% (30). Informed consent was obtained from each participant; and their results were confidential.

**Statistical analysis**

All data were transferred directly into SPSS (version 15). For data analysis, Chi-square and “t-test” were used and level of confidence interval was 95%. 

## Results

Three hundred questionnaires were completed by randomly selected 150 infertile couples. The mean age of women was 28.3 (between 20-42) and for men 32.4 (between 22-46) years. Twenty one of 300 (7%) were illiterate and 58 of them (19.3%) were educated, 132 (44%) completed the high school and 92 (30.6%) had higher education. The more frequent ethnicity of the couples was Fars (82.3%) and then Lore (7.3%). The worse health score was seen in Arab people and the best score was for Lore people. The mean grade scores of total GHQ for Arabs was 33.33±17.16 and for Lore was 14.82±6.79. 

The difference between them was significant (p<0.001). The scores of all of sub- scales of GHQ in women were more than men. The mean total score of the general health of women was more than men. The mean grade scores of GHQ for males was 17.5±9.97 and for females 22.92±13. The relation between the sex and GHQ in total and all of sub- scales of GHQ was significantly different (p=0.001). There is no significant difference between the duration of infertility and mean grade scores of sub- scales of GHQ and GHQ in total. However, with increasing the duration of infertility, the mean grade scores of GHQ increased (Table II). 

The mean grade scores of all of sub- scales of GHQ in participants with university education were lower than others. There is significant difference between the somatic symptoms and education of participants (p=0.046). There is relationship between the health situation of participants and their economic levels; worse health condition was seen in lower economic level. The mean grade scores of GHQ of high economic level were 16.8±9.8 and for participants with low economic levels was 28.21±13.56 (Table IV). There is significant difference between the economic level of participants and their GHQ (p=0.008). 

The mean grade scores of participants in age of 25-29 years old (22.79±12.35) is more than others. There is significant difference between the age of participants and their GHQ scores (p=0.005). The mean grade scores of participants in Lores (14.82±6.79) is lower than others. There is significant difference between the different ethnicity of participants and their GHQ scores (p=0.004).

**Table I:** The mean sub- scales grade scores of GHQ based on sex.

** Sex **	**Male** **Mean (±SD)**	**Female** **Mean (± SD)**	**p-value**
**Sub- scales**			
Somatic symptoms	4.17 (± 3.02)	5.88 (± 3.9)	0.000 1
Anxiety insomnia	4.95 (± 3.73)	6.45 (± 4.92)	0.09
Social dysfunction	6.31(± 2.48)	7.39 (± 2.64)	0.0001
Severe depression	2.07 (± 3.39)	3.24 (± 4.06)	0.001
Total	17.5 (± 9.97)	22.92 (± 13)	0.0001

The results showed women are worse than men in each and total scores significantly.

**Table II:** The mean sub- scales grade scores of GHQ based on duration of marriage of cases

** Duration of marriage**	**<5 years** **Mean(± SD)**	**5-10 years** **Mean (± SD)**	**>10 years** **Mean(± SD)**	**p-value**
**Sub – scales**				
Somatic symptoms	5.19 (± 3.52)	4.69 (±4.1)	5.59 (± 4.41)	0.521
Anxiety insomnia	5.42 (±4.3)	5.68 (± 4.28)	6.21(± 5.19)	0.845
Social dysfunction	7.06 (±2.44)	6.71 (±2.59)	7.83 (± 2.95)	0.179
Severe depression	2.13 (± 3.32)	2.82 (± 3.82)	3.15(± 4.33)	0.278
Total	19.8 (± 10.9)	19.84 (± 11.29)	21.78(± 14.65)	0.936

There is no relation between scores and duration of infertility. Higher scores were seen in longer duration, which are not significant.

**Table III:** The mean sub- scales grade scores of GHQ based on education of cases

** Education**	**Illiterate and lower** **Mean(± SD)**	**Secondary school** **Mean(± SD)**	**High school** **Mean(± SD)**	**University** **Mean(± SD)**	**p-value**
**Sub – scales**					
Somatic symptoms	5.19 (± 3.4)	5.71(± 4.07)	5.4 (± 3.87)	4.0 (± 2.62)	0.046
Anxiety insomnia	5.36(±3.72)	6.11(± 4.71)	5.98(± 4.75)	5.12 (± 3.86)	0.706
Social dysfunction	6.62 (± 1.32)	6.65 (± 2.96)	6.95 (± 2.69)	6.58(± 2.53)	0.573
Severe depression	2.95 (± 3.47)	3.09 (± 4.33)	2.68(± 3.74)	2.3 (± 3.58)	0.662

The somatic symptoms have a significant relation with the education of the cases.

**Table IV:** The mean grade scores of GHQ in infertile couples in relation with their economic level

**Economic level**	**No**	**Mean**	**SD**
Good	5	16.8	3.96
Rather good	14	16.43	9.97
Moderate	213	19.1	10.42
Low	48	23.42	14.79
Poor	19	28.21	13.56
Total	299	20.21	11.88

p=0.008

This table showed couples with higher economic level have better grade of general health according to GHQ test.

**Table V:** Distribution and mean grade scores of GHQ in infertile couples in relation with their age

**Age (years)**	**No**	**Mean**	**SD**
20-24	37	21.73	13.11
25-29	104	22.79	12.35
30-34	89	18.19	10.65
35+	68	19.19	11.37
Total	298	20.21	11.88

p=0.005

There is significant relation between the age and GHQ scores of the infertile couples.

**Table VI:** The mean grade scores of GHQ in infertile couples in relation with their ethnicity.

**Ethnic **	**No**	**Mean**	**SD**
Turkish	11	26.1	10.33
Lore	22	14.82	6.79
Fars	247	19.97	11.77
Others	20	26.15	15.04
Total	300	20.21	11.88

p=0.004

This table showed the ethnicity of Iranian patients affect their general health.

## Discussion

This research using GHQ-28 questionnaire investigated health situation of 300 infertile men and women planning to undergo IVF or ICSI treatment. The data of this study showed the scores of all of sub- scales of GHQ in women are more than men. Previous studies have revealed that the effect of infertility in women is higher than men (5-11, 31, 32), and women with planning IVF experienced have more emotional and social problems than women of same age groups in the general population (19). 

The difference of the GHQ-28 scale due to gender was also found by Goldberg and Williams (25). Women usually have higher score on the GHQ-28 scale than men (25). Other studies have suggested that impact of infertility and its treatment is higher in women than in men (33-35). Fekkes *et al* demonstrated that having children was more important for women than men (34). Results from the present study about gender are in line with the previous conclusions. Shindel *et al* revealed that depression, social dysfunction and sexual problems were more frequent among male partners of infertile couples than fertile men. In addition, they significantly are in lower mental health score than fertile men (35).

In present study severe depression in male was 2.07±3.39 and in women 3.24±4.06. These data is confirmed by the result of Alizadegan *et al*, which described that 48% of infertile women and 23.8% of infertile men had depression, but 5.3% of women and 2.5% men in population are severly depressed (36). 

There is no significant difference between the duration of infertility and mean grade scores of sub-scales of GHQ and GHQ in total. Ragni *et al* in Italy showed duration of infertility dose significantly affect the psychological area of infertile couples and it might be reflected by the duration of infertility. However, it does not markedly affect subjective health status in patients requiring IVF (37). In our study the higher score in social dysfunction scale observed in patients with infertility duration of more than 10 years, while this was more than 5 years in the Italian patients with infertility (37). However, Rashidi *et al* found quality of life, physical or mental; have no relation with either infertility duration or cause of infertility (38).

In this study the situation of health are worse in younger participants and it goes better with older age. Matsubayashi *et al* (41), Beutel *et al* (42) and Rashidi *et al* (38) found the same results, which showed younger patients significantly have worse health condition. However Guz *et al* (39) and Khayata *et al* (40) argued that psycho-cognitive sign of anxiety and depression have a significant relationship with old age. 

Our study finding showed that mean grade scores of all of sub-scales of GHQ in higher educated couples is lower than others. However the difference was not significant. In addition, it revealed that socio- demographic characteristics of infertile couples could be important for their health situation. These results are same as the results of study by Brkovich *et al* (9). 

The result of present study is in line of Hus and Kuo (43), they argued that health situation of infertile couples vary according to their level of education. In the present study severe depression was found in secondary school education and social dysfunction in high school education.

This investigation showed the worse health score was found in couples with lower income. The relationship between income and health situation was significantly different (p=0.008). Schmidt *et al* (44) confirmed that psychological situation among low income couples is worse than couples with high income.

In conclusion, the study finding suggest that the infertility have significant effect on health situation of infertile couples, specially infertile women, which are at risk of somatic symptoms, anxiety, insomnia, social dysfunction, and severe depression. General health evaluation using GHQ should be provided to help and support in order to improve the infertile couples, health situation.

## References

[1] Evers JL (2002). Female sub fertility. Lancet.

[2] Abbasi-Shavazi MJ, Razeghi Nasrabad HB, Behjati Ardekani Z, Akhondi MA (2006). Attitudes of infertile women towards gamete donation: a case study in Tehran. J Reprod Infertil.

[3] Leiblum Sr, Greenfield DA, Leiblum SR (1997). Infertility. psychological issues and counseling strategies.

[4] Fassino S, Piero A, Boggio S, Piccioni V, Garzaro L (2002). Anxiety, depression and anger suppression in infertile couples. Hum Reprod.

[5] Chen TH, Chang SP, Tsai CF, Juang KD (2004). Prevalence of depressive and anxiety disorders in an assisted reproductive technique clinic. Hum Reprod.

[6] Greil AL (1997). Infertility and psychological distress: a critical review of the literature. Social Sci Med.

[7] Mohammadi MR, Ghalagabadi Farahani F (2001). Emotional and Mental problems of infertility and methods for contrast with them. J Reprod Infertil.

[8] Leiblum SR, Greenfield DA, Leiblum, S (1997). The course of infertility: immediate and long-term reactions. Infertility: Psychological Issues and Counseling Strategies.

[9] Brkovich AM, Fisher WA (1998). Psychological distress and infertility: forty years of research. J Psychosom ObstetGynaecol.

[10] Burns LH, Covington SH, Burns LH, Covington SH (1999). Psychology of Infertility. Infertility Counseling. A Comprehensive Handbook for Clinicians.

[11] Berg BJ, Wilson JF (1991). Psychological sequelae of infertility treatment: the role of gender and sex-role identification. Soc Sci Med.

[12] Wright J, Duchesne C, Sabourin S, Bissonnette F, Benoit J, Girard Y (1991). Psychosocial distress and infertility: men and women respond differently. Fertil Steril.

[13] Daniluk JC, Leiblum, S (1997). Gender and infertility. Infertility: Psychological Issues and Counseling Strategies.

[14] Nachtigall RD, Becker G, Wozny M (1992). The effects of gender-specific diagnosis on men's and women's response to infertility. Fertil Steril.

[15] Jorda C, Revenson TA (1999). Gender differences in coping with infertility: a meta-analysis. J Behav Med.

[16] O'Moore AM, O'Moore RR, Harrison RF, Murphy G, Carruthers ME (1983). Psychosomatic aspects in idiopathic infertility: effects of treatment with autogenic training. J Psychosom Res.

[17] Callan VJ (1987). The personal and marital adjustment of mothers and of voluntarily and involuntarily childless wives. JMF.

[18] Callan VJ, Hennessey JF (1998). The psychological adjustment of women experiencing infertility. Br J Me Psychol.

[19] Fekkes M, Buitendijk SE, Verrips GH, Braat DD, Brewaeys AM, Dolfing JG (2003). Health-related quality of life in relation to gender and age in couples planning IVF treatment. Hum Reprod.

[20] Blalock SJ, Devellis RF, Brown GK, Wallston KA (1989). Validity of the center for epidemiological studies depression scale in arthritis population. Arthritis Rheum.

[21] Pincus T, Callahan LF (1993). Depression scale in rheumatoid arthritis: criterion contamination of patient responses. Patient EDUC Couns.

[22] Krol B, Sanderman R, Moun T, Suurmeijer T (1993). Social support, rheumatoid arthritis and quality of life: concept, measurement and research. Patients Educ Couns.

[23] Goldberg DP, Hillier VF (1979). A scaled version of the General Health Questionnaire. Psychol Med.

[24] Krol B, Sanderman R, Moun T, Suurmeijer T, Doeglas D, Krijnen W (1994). A comparison of General Health Questionnaire- 28 between patients with rheumatoid arthritis from the Netherland, France, Sweden and Norway. Eur J Psychol Assess.

[25] Goldberg DP, Williams P (1988). A user's guide to the General Health Questionnaire.

[26] Mc Dowell I, Newell C (1987). Measuring Health.

[27] Banks M (1983). Validitation of the General Health Questionnaire in a young community sample. Psychol Med.

[28] Sanderman R, Stewart R (1990). The assessment of psychological distress: psychometric properties of the GHQ. Int J Health Sci.

[29] Yaghoby N (1996). A survey about epidemiology of psychotic disorders in Soumeah sara city. Thought and Behavior in ClinicalPsychology.

[30] Torkamani por Rouh angiz, Palahang H (1999). Prevalence of mental disorders in the internal ward of Kashani Hospital, Shahrekord. J Shahrekord Univ Med Sci.

[31] Van Balen F, Trimbos-Kemper TC (1994). Factors influencing the well-being of long-term infertile couples. J Psychosom Obstet Gynaecol.

[32] Pasch LA, Dunkel-Schetter C, Christensen A (2002). Differences between husbands, and wives, approach to infertility affect marital communication and adjustment. Fertil Steril.

[33] Lee TY, Sun GH, Chao SC (2001). The effect of an infertility diagnosis on the distress, marital and sexual satisfaction between husbands and wives in Taiwan. Hum Repord.

[34] Fekkes M, Buitendijk SE, Verrips GH, Braat DD, Brewaeys AM, Dolfing JG (2003). Health related quality of life in relation to gender and age in couples planning IVF treatment. Hum Repord.

[35] Shindel AW, Nelson CJ, Naughton CK, Ohebshalom M, Mulhall JP (2008). Sexual function and quality of life in the male partner of infertile couples: prevalence and correlates of dysfunction. J Urol.

[36] Alizadegan S, Ashrafi M, Baghestani AR (2005). Mental Health Status of Women Referring to Royan Infertility Treatment Center. Fertil Steril.

[37] Ragni G, Mosconi P, Baldini MP, Somigliana E, Vegetti W, Caliari I (2005). Health- related quality of life and need for IVF in 1000 Italian infertile couples. Hum Reprod.

[38] Rashidi B, Montazeri A, Ramezanzadeh F, Shariat M, Abdinia M, Ashrafi M (2008). Health- related quality of life in infertile couples receiving IVF or ICSI treatment. BMC Health Services Research.

[39] Guz H, Ozkan A, Sarisoy G, Yanik F, Yanik A (2003). Psychiatric symptoms in Turkish infertile women. J Psychosom Obstet Gynecol.

[40] Khayata GM, Rizk DE, Hasan MY, Ghazal Aswad S, Asaad MA (2003). Factors influencing the quality of life of infertile women in United Arab Emirates. Int J Gynecol Obstet.

[41] Matsubayashi H, Hosaka T, Izumi T, Makino T (2001). Emotional distress of infertile women in Japan. Hum Reprod.

[42] Beutel M, Kupfer J, Kirchmeyer P, Kehde S, Kohn FM, Schroeder- printzen I (1999). Treatment- related stresses and depression in couples undergoing assisted reproduction treatment by IVF or ICSI. Andrologia.

[43] Hsu YL, Kuo BJ (2002). Emotional reactions and coping behaviors as well as correlated factors for infertile couples receiving assisted reproductive technologies. J Nurs Res.

[44] Schmidt L, Holstien BE, Boivin J, Tjqrnhqj-Thomsen T, Blaabjerg J, Hald F (2003). High rating of satisfaction with fertility treatment is common: finding from the Copenhagen multi-center psychological infertility(COMPI) Research programe. Hum Repord.

